# CMR right ventricular strain assessment using feature tracking cine images: agreement with echocardiography

**DOI:** 10.1186/1532-429X-14-S1-P244

**Published:** 2012-02-01

**Authors:** Daniel Augustine, Joseph J Suttie, Peter Cox, Adam J Lewandowski, Cameron Holloway, Steffen E Petersen, Saul Myerson, Stefan Neubauer, Paul Leeson

**Affiliations:** 1 Clinical Cardiovascular Research Facility, University Of Oxford, Oxford, UK; 2 Centre for Clinical Magnetic Resonance Research, University of Oxford, Oxford, UK; 3 William Harvey Research Institute, William Harvey Research Institute, Barts and the London NIHR Cardiovascular Biomedical Research Unit, London, UK

## Summary

Right ventricular strain assessment by feature tracking from cine images shows acceptable levels of agreement with echocardiography

## Background

RV ejection fraction is the recognised marker of RV function although the assessment of deformation by strain analysis provides potential means for assessing early ventricular dysfunction before other recognised markers fall.

RV strain analysis by tagging is time consuming and requires significant operator expertise due to the nature of the RV anatomy (particularly the thin free wall and increased trabeculations). The potential to analyse RV strain from cine images using semi automated software to track the endocardial border now exists, feature tracking ‘FT’ (TomTec Diogenes, Germany). Our aims are to:

1. Assess the agreement between FT RV HLA view and 2D echocardiography (2DE) for global peak RV free wall longitudinal strain

2. To assess the correlation between FT derived basal peak systolic velocity and basal displacement with common markers of echocardiographic RV longitudinal performance (peak systolic S wave by tissue Doppler and tricuspid annular plane systolic excursion, TAPSE).

## Methods

19 individuals underwent 3T CMR (Siemens, Germany) to obtain RV HLA cine images. Apical 4 chamber views to assess the RV free wall by 2D echocardiography was performed using Toshiba Artida (2.5MHz PST-25SX probe).

Post processing assessment was carried out using TomTec Diogenes (for FT) and TomTec Cardiac Performance Analysis (for 2DE)

Data is presented as mean ± SD and agreement assessed using Bland Altman analysis and Pearsons Correlation.

## Results

Heart rates during CMR (64.8 ± 8.8) and 2DE (63.4 ± 12.2) were similar (P = 0.64). The temporal resolution of 2DE (16.5 ± 1.2ms) was shorter than that for CMR (37.8 ± 4.2ms), P<0.05. The analysis time needed for FT HLA was 220 ± 40s.

Results are presented in Table 1. Acceptable bias was seen when comparing FT HLA with 2DE. 11 out of 57 RV FT HLA free wall segments (19.29%) were excluded from the analysis due to poor tracking.

**Table 1:** Results

Variable	FT HLA Mean ± SD	2D Echo	FT HLA vs. 2D Echo		
	
			Bias	LOA	95% CI
Longitudinal Strain (%)	-21.1 ± 2.1	-18.7 ± 2.0	-2.33	-9.4 to 4.7	4.1 to -0.4

FT: Feature Tracking; HLA: Horizontal Long Axis; SD: Standard Deviation; LOA: Limits Of Agreement; CI: Confidence Interval.

No significant correlations were seen between FT and 2DE for peak basal velocity or peak basal displacement.

## Conclusions

FT allows the assessment of RV strain from cine images and shows acceptable levels of agreement with 2DE. The lack of correlation between FT velocities and displacement values when compared with 2DE is likely due to a combination of the improved temporal resolution of 2DE together with the precise locations for the FT tracking measurements not being known.

The ease of analysis of RV free wall FT is promising although further insights into the algorithms used are needed to make more accurate judgements as to the agreement with echocardiography, to define normal values and to assess its use in disease populations with RV dysfunction.

## Funding

The research is funded by the Engineering and Physical Sciences Research Council (EPSRC) grant EP/G030693/1.

**Figure 1:**
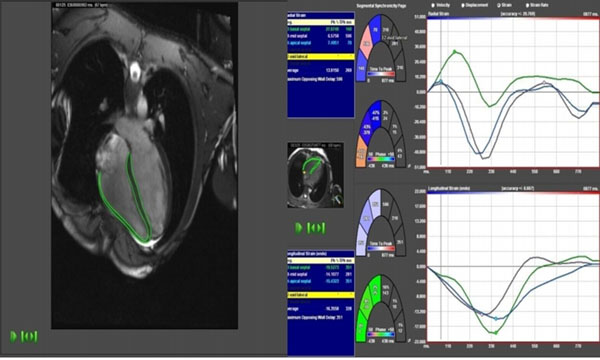
Left: FT strain assessment - RV endocardial and epicardial borders delineated from CMR cine image; Right: RV free wall strain curves, lower graph (TomTec Diogenes, Germany)

